# Potential efficacy of hepatic arterial infusion chemotherapy using gemcitabine, cisplatin, and 5-fluorouracil for intrahepatic cholangiocarcinoma

**DOI:** 10.1371/journal.pone.0266707

**Published:** 2022-04-22

**Authors:** Masatsugu Ishii, Osamu Itano, Jun Morinaga, Hirofumi Shirakawa, Satoshi Itano

**Affiliations:** 1 Department of Gastroenterology, Kurume Chuo Hospital, Fukuoka, Japan; 2 Department of Hepato-Biliary-Pancreatic Surgery, Tochigi Cancer Center, Tochigi, Japan; 3 Department of Hepato-Biliary-Pancreatic and Gastrointestinal Surgery, School of Medicine, International University of Health and Welfare, Chiba, Japan; 4 Department of Clinical Investigation, Kumamoto University Hospital, Kumamoto, Japan; Texas A&M University, UNITED STATES

## Abstract

Intrahepatic cholangiocarcinoma (ICC) has a poor prognosis, as the resectability rate is low due to its diagnosis at a late/advanced stage. Moreover, most patients with resected ICC eventually relapse. Hepatic arterial infusion chemotherapy (HAIC) has been indicated only by a few reports to be effective in patients with advanced ICC; thus, its efficacy for these patients remains unclear. This study aimed to evaluate the efficacy of HAIC using gemcitabine, cisplatin, and 5-fluorouracil in patients with advanced ICC. A total of 18 patients who underwent HAIC were retrospectively investigated. The patients received gemcitabine, cisplatin, and 5-fluorouracil through one artery. In patients who received gemcitabine plus cisplatin (*n* = 10), the response and disease control rates were 0% and 80.0%, respectively; the median overall survival (OS) and progression-free survival (PFS) after treatment initiation were 6.3 and 3.7 months, respectively. In patients who never received chemotherapy (*n* = 8), the response and disease control rates were 37.5% and 75%, respectively; the median OS and PFS were 20.6 and 8.1 months, respectively. Moreover, we compared the patients who received HAIC using gemcitabine, cisplatin, and 5-fluorouracil to patients whose tumors were refractory to systemic gemcitabine and cisplatin therapy. The OS of the patients who received HAIC was better than that of the patients who received standard chemotherapy cohort since the gemcitabine plus cisplatin combination therapy-refractory response and disease onset (*P* = 0.045, 0.006). HAIC using gemcitabine, cisplatin, and 5-fluorouracil may be effective as a therapeutic option for patients with advanced ICC.

## Introduction

The incidence of intrahepatic cholangiocarcinoma (ICC) has increased over the recent years [1]. Surgical resection remains the only potentially curative option for patients with advanced ICC. Nonetheless, the resectability rate remains low, as ICC is generally diagnosed at an advanced stage [2]. Additionally, most patients with resected ICC eventually relapse.

Systemic chemotherapy for biliary tract cancer (BTC) has been investigated, and gemcitabine plus cisplatin combination therapy (GC) has been indicated by some reports to significantly prolong overall survival (OS) in patients with BTC [3, 4]. GC is accepted as the standard first-line treatment of advanced BTC in many countries. However, the optimal chemotherapy regimen for patients with advanced BTC refractory to GC has not yet been established.

Hepatic arterial infusion chemotherapy (HAIC) is an effective treatment for unresectable hepatocellular carcinoma in Japan [5]. Some studies have reported that HAIC is a safe and enhanced drug delivery method for tumors [6, 7]. Although it is not a common treatment for ICC worldwide, a few reports have indicated that HAIC is effective in patients with advanced ICC [8, 9]. However, these studies reported on patients with HAIC receiving only 5-fluorouracil. In several previous studies, gemcitabine and platinum were the key drugs for ICC [3, 4, 10]. In some studies, HAIC using gemcitabine for patients with advanced ICC yielded favorable results [11, 12]. Therefore, HAIC using gemcitabine, cisplatin, and 5-fluorouracil (GEM-FP) may offer favorable results in patients with advanced ICC. This study aimed to evaluate the efficacy of HAIC using GEM-FP in patients with advanced ICC.

## Materials and methods

### Ethics statements

This study was conducted in accordance with the principles outlined in the Declaration of Helsinki and was approved by the ethics committee of our hospitals [20210001 (Kurume Chuo Hospital), 21-C005 (Tochigi Cancer Center)]. Written informed consent was obtained from all patients.

### Patients

Overall, 18 patients who received HAIC at Kurume Chuo Hospital between April 2014 and December 2020 were recruited for this study. This study followed the Strengthening the Reporting of Observational Studies in Epidemiology (STROBE) reporting guidelines for observational studies [13]. The inclusion criteria for HAIC were as follows: age ≥20 years; Eastern Cooperative Oncology Group performance status of 0 or 1; adequate oral intake capacity; adequate bone marrow function (white blood cell count ≥3000/mm^3^, neutrophil count ≥1500/mm^3^, platelet count ≥100,000/mm^3^, hemoglobin level ≥8.0 g/dL), serum total bilirubin level ≤2.0 mg/dL (or ≤3.0 mg/dL under biliary drainage), adequate renal function (serum creatinine level ≤1.5 mg/dL and creatinine clearance or estimated glomerular filtration rate by Cockcroft-Gault formula ≥60 mL/min), and adequate nutrition status (serum albumin level ≥3.0 g/dL); no abnormal electrocardiogram findings within 28 days before registration; and written informed consent. The exclusion criteria for HAIC were extrahepatic bile duct cancer (*n* = 2) and previous chemotherapy other than GC (*n* = 2).

**Fig 1:**
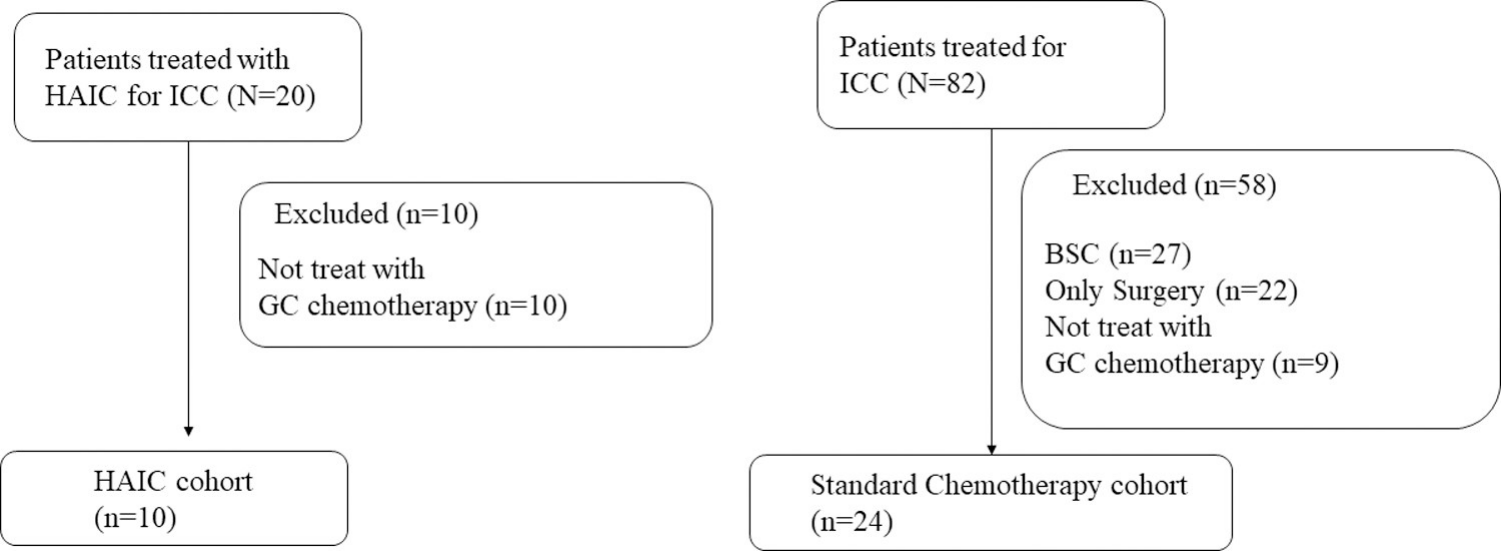
Patient classification.

To evaluate the safety and efficacy of HAIC using GEM-FP, we compared these patients with HAIC to those with advanced ICC who underwent GC chemotherapy at Tochigi Cancer Center between April 2011 and August 2021 (Fig 1).

### Data collection and treatment regimen

Patient data were extracted from the hospital database. The diagnosis of ICC was established based on typical imaging features or pathological findings. We performed computed tomography (CT) during hepatic arteriography using a catheter in the hepatic artery. The artery supplying blood to the tumor and the plural arteries supplying blood to the tumor were identified. We performed HAIC using a delivery port system or a new indwelling catheter system (System-i) [14]. We administered gemcitabine (600 mg over 1 hour), cisplatin (20 mg over 1 hour), and 5-fluorouracil (1000 mg, 46-h constant infusion) through one artery on day 1. If the plural arteries were found to be supplying blood to the tumor on day 3, we administered the same drugs at the same dosage through a different artery. If the patients had not received systemic GC or previous GC chemotherapy responded better than SD in metastatic lesion other than the liver, they simultaneously received a systemic infusion with gemcitabine (500 mg over 30 min), cisplatin (20 mg over 1 hour), and 5-fluorouracil (1000 mg, 46-h constant infusion) (GCF) on day 1. We adjusted the hepatic arterial and systemic doses of gemcitabine to avoid exceeding a total dose of 1000 mg. The dose of gemcitabine was determined with reference to that used in previous studies [11, 12]. The drug dose was changed according to the patient’s performance status, age, and presence of distance metastasis. We used this regimen once every three weeks.

### Treatment evaluation

The primary endpoint was OS. Survival time was calculated from the day of starting HAIC, with deaths from all causes treated as events. The secondary endpoints were progression-free survival (PFS) and adverse effects. As a rule, patients were monitored monthly for disease recurrence; this overall assessment included a physical examination, radiography, ultrasonography, CT, and laboratory examinations. Tumor response to this therapy was assessed per RECIST version 1.1 using CT or magnetic response imaging. Adverse drug reactions were assessed according to the Common Terminology Criteria for Adverse Events version 5.0 [15].

### Statistical analysis

Continuous variables were compared using the Mann–Whitney U test. Time to PFS and OS were estimated using the Kaplan–Meier method. All statistical analyses were performed using IBM SPSS Statistics for Windows/Macintosh version 27 (IBM Corp., Armonk, NY, USA) with *P*<0.05 considered as statistically significant.

## Results

### Patient characteristics

A total of 18 patients with advanced ICC received HAIC using GEM-FP between August 2014 and December 2020. Table 1 summarizes the patient characteristics. The population consisted of 11 men and 7 women, ranging in age from 36 to 78 years. Among the patients, there were six smokers (33.3%), five alcohol drinkers (27.8%), no patient with hepatitis B antigen, two patients with hepatitis C antibody (11.1%), one patient with diabetes mellitus (5.6%), three patients whose body mass index was >25 kg/m^2^ (16.7%), and one patient with primary sclerosing cholangitis (5.6%). Ten patients (55.6%) underwent GC chemotherapy, whereas eight patients (44.4%) had never undergone chemotherapy; among the latter, there were five patients (62.5%) who received additional systemic GC chemotherapy. There were two patients (20%) who received additional systemic GC chemotherapy among the patients who underwent HAIC using GEM-FP after GC chemotherapy. There was no difference in background factors between the two groups.

**Table 1:** Patient characteristics of 18 patients who received HAIC using GEM-FP.

		All (n = 18)	No primary chemotherapy (n = 8)	After GC chemotherapy (n = 10)	*P*
Age		68.5 (36–78)	68.5 (64–78)	63 (36–78)	0.408
Sex	Male	11 (61.1)	6 (75)	5 (50)	0.280
	Female	7 (38.9)	2 (25)	5 (50)	
Smoking	Yes	6 (33.3)	3 (37.5)	3 (30)	0.737
	No	12 (66.7)	5 (62.5)	7 (70)	
Alcohol consumption	Yes	5 (27.8)	3 (37.5)	2 (20)	0.410
	No	13 (72.2)	5 (62.5)	8 (80)	
HCV	Yes	2 (11.1)	1 (12.5)	1 (10)	0.867
	No	16 (88.9)	7 (87.5)	9 (90)	
Diabetes mellitus	Yes	1 (5.6)	1 (12.5)	0 (0)	0.250
	No	17 (94.4)	7 (87.5)	10 (100)	
Obesity (BMI>25 kg/m^2^)	Yes	3 (16.7)	1 (12.5)	2 (20)	0.671
	No	15 (83.3)	7 (87.5)	8 (80)	
Primary sclerosing cholangitis	Yes	1 (5.6)	0 (0)	1 (10)	0.357
	No	17 (94.4)	8 (100)	9 (90)	
Primary chemotherapy	Gemcitabine	4 (22.2)	0 (0)	4 (40)	
	Gemcitabine+ cisplatin	10 (55.6)	0	10 (100)	
	Gemcitabine + S-1	2 (11.1)	0	2 (20)	
	S-1	3 (16.7)	0	3 (30)	
	FOLFIRINOX	1 (5.6)	0	1 (10)	
Tumor number	Unifocal	6 (33.3)	4 (50)	2 (20)	0.180
	Multifocal	12 (66.7)	4(50)	8 (80)	
Maximum diameter (mm)		55 (17–170)	55 (17–170)	47.5 (10–90)	0.829
CEA (ng/mL)		6.55 (1–929)	28.3 (1.9–929)	5.05 (1–342)	0.274
CA19-9 (mAU/mL)		65.2 (2.2–5016)	65.2 (4.1–5016)	71.9 (2.2–1952)	0.633
Distant metastasis (without liver)	Yes	12 (66.7)	6 (75)	6 (60)	0.502
	No	6 (33.3)	2 (25)	4 (40)	
Metastatic organ	Liver	9 (50)	3 (37.5)	6 (60)	
	Lung	4 (22.2)	3 (37.5)	1 (10)	
	Lymph node	9 (50)	2 (25)	7 (70)	
	Peritoneum	1 (5.6)	1 (12.5)	0 (0)	
	Bone	3 (16.7)	2 (25)	1 (10)	
Additional systemic GCF chemotherapy	Yes	7 (38.9)	5 (62.5)	2 (20)	0.066
	No	11 (61.1)	3 (37.5)	8 (80)	

Data are expressed as median (range) or n (%)

CEA: carcinoembryonic antigen, CA19-9: carbohydrate antigen 19–9, FOLFIRINOX: 5-fluorouracil, folinic acid, irinotecan, and oxaliplatin combination therapy, GCF: gemcitabine, cisplatin, and 5-fluorouracil combination therapy, HAIC: hepatic arterial infusion chemotherapy, HCV: hepatitis C virus.

A total of 24 patients with advanced ICC received GC between April 2011 and August 2021 at Tochigi Cancer Center. Table 2 summarizes the patient characteristics. The standard chemotherapy cohort consisted of patients with tumors refractory to GC therapy. The population consisted of 11 men and 13 women, ranging in age from 31 to 79 years. Among the patients, there were 14 smokers (58.3%), 13 alcohol drinkers (54.2%), one patient with hepatitis B antigen (4.2%), three patients with hepatitis C antibody (12.5%), seven patients with diabetes mellitus (29.2%), five patients with a body mass index of >25 kg/m^2^ (20.8%), and no patient with primary sclerosing cholangitis. There was no difference in background factors between the HAIC and standard chemotherapy cohorts.

**Table 2:** Patient characteristics in the HAIC and standard chemotherapy cohorts.

		All (n = 34)	HAIC cohort (n = 10)	Standard chemotherapy cohort (n = 24)	*P*
Age		64 (31–79)	63 (36–78)	64 (31–79)	0.897
Sex	Male	16 (47.1)	5 (50)	11 (45.8)	0.824
	Female	18 (52.9)	5 (50)	13 (54.2)	
Smoking	Yes	17 (50)	3 (30)	14 (58.3)	0.132
	No	17 (50)	7 (70)	10 (41.7)	
Alcohol consumption	Yes	15 (44.1)	2 (20)	13 (54.2)	0.068
	No	19 (55.9)	8 (80)	11 (45.8)	
HBS antigen	Yes	1 (2.9)	0 (0)	1 (4.2)	0.512
	No	33 (97.1)	10 (100)	23(95.8)	
HCV	Yes	4 (11.8)	1 (10)	3 (12.5)	0.837
	No	30 (88.2)	9 (90)	21 (87.5)	
Diabetes mellitus	Yes	7 (20.6)	0 (0)	7 (29.2)	0.055
	No	27 (79.4)	10 (100)	17 (70.8)	
Obesity (BMI>25 kg/m^2^)	Yes	7(20.6)	2 (20)	5 (20.8)	0.956
	No	27 (79.4)	8 (80)	19 (79.2)	
Primary sclerosing cholangitis	Yes	1 (2.9)	1 (10)	0 (0)	0.116
	No	33 (97.1)	9 (90)	24 (100)	
Tumor number	Unifocal	8 (23.5)	2 (20)	6(25)	0.754
	Multifocal	26 (76.5)	8(80)	18 (75)	
Maximum diameter (mm)		57.7 (10–121.77)	47.5 (10–90)	57.7 (15.79–121.77)	0.515
CEA (ng/mL)		5 (0.9–1969.4)	5.05 (1–342)	4.75 (0.9–1969.4)	0.838
CA19-9 (mAU/mL)		125.04 (2–96093.06)	71.9 (2.2–1952)	173.2 (2–969093.06)	0.401
Distant metastasis (without liver)	Yes	23 (67.6)	6 (60)	17 (70.8)	0.538
	No	11 (32.4)	4 (40)	7 (29.2)	
Metastatic organ	Liver	20 (58.8)	6 (60)	14 (58.3)	
	Lung	8 (23.5)	1 (10)	7 (29.2)	
	Lymph node	15 (44.1)	7 (70)	8 (33.3)	
	peritoneum	9 (26.5)	0 (0)	9 (37.5)	
	Bone	3 (8.8)	1 (10)	2 (5.9)	

Data are expressed as median (range) or n (%)

CEA: carcinoembryonic antigen, CA19-9: carbohydrate antigen 19–9, HAIC: hepatic arterial infusion chemotherapy, HBS: hepatitis B antigen, HCV: hepatitis C virus.

### Effect and survival

**Fig 2:**
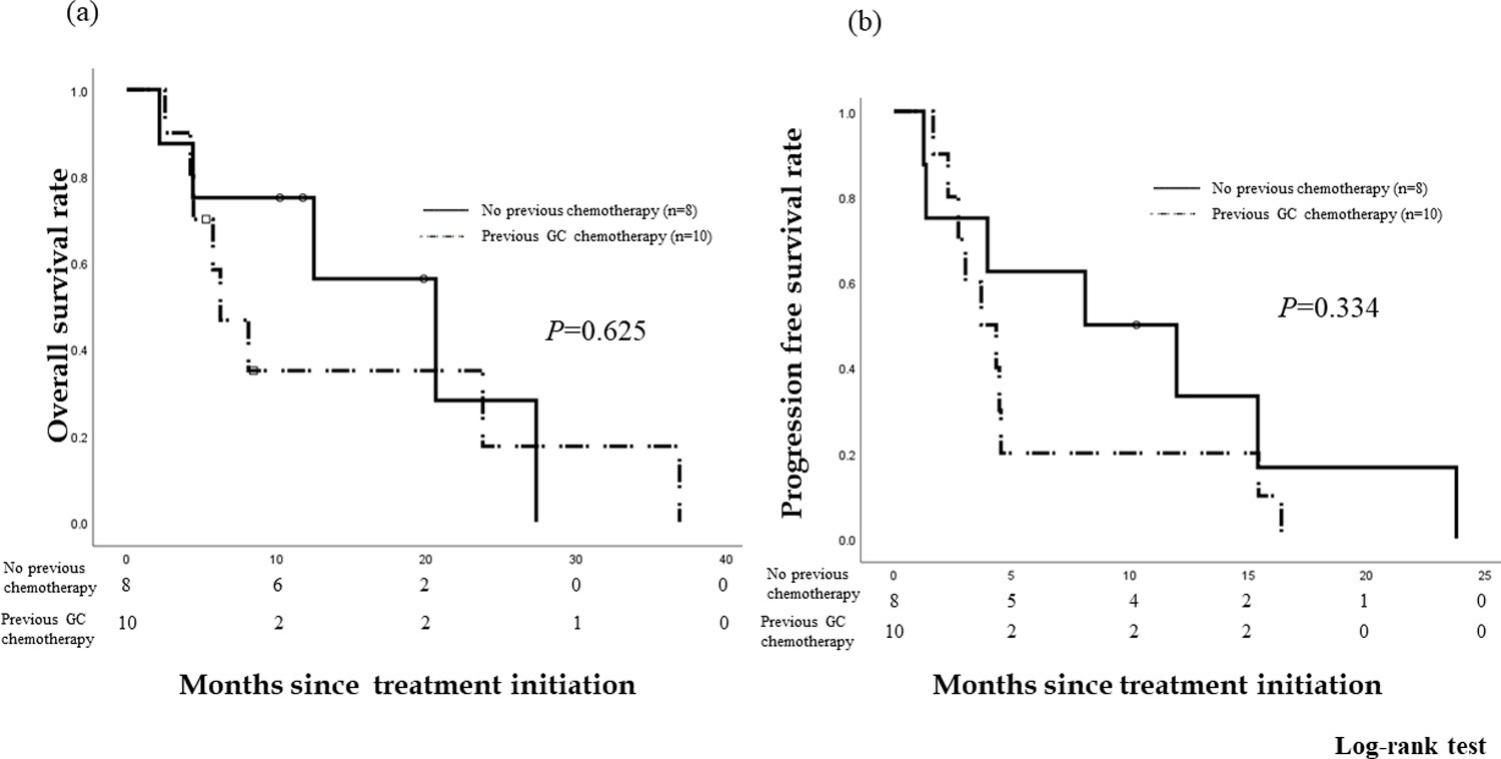
Overall survival (OS) and progression-free survival (PFS) rates in the patients who received no previous chemotherapy vs the patients who received GC chemotherapy. The OS rates were 75.0% and 58.3% at 6 months and 75.0% and 35.0% at 1 year in patients who received no previous chemotherapy and those who received GC chemotherapy, respectively. The median OS was 20.6 months (95% *confidence interval* [*CI*] 8.03–33.23) and 6.3 months (95% *CI* 2.97–9.56), respectively. The PFS rates were 62.5% and 20.0% at 6 months and 33.3% and 20.0% at 1 year), respectively. The median PFS was 8.1 months (95% *CI* 0.00–17.6) and 3.7 months (95% *CI* 1.69–5.71), respectively. There was no significant difference in the OS and PFS since treatment initiation between patients who underwent no chemotherapy and those who underwent GC (*P* = 0.625 and *P =* 0.334, respectively).

The median follow-up period in the patients was 8.3 months (range: 2–36 months) after treatment initiation. Of the 18 patients, eight patients who had not undergone a previous chemotherapy and ten patients who underwent GC chemotherapy were evaluated. Among the patients who had not undergone previous chemotherapy, no complete response (CR) was observed. Three patients (37.5%) showed a partial response (PR), while three patients (37.5%) had stable disease (SD). The response rate (CR + PR) was 37.5%. The disease control rate (CR + PR + SD) was 75.0%. The OS rates were 75.0% at 6 months and 75.0% at 1 year. The median OS was 20.6 months (95% *confidence interval* [*CI*] 8.03–33.23). The PFS rates were 62.5% at 6 months and 33.3% at 1 year. The median PFS was 8.1 months (95% *CI* 0.00–17.6) (Fig 2).

Ten patients who received GC were evaluated. No CR or PR was observed. Eight patients (80.0%) had SD. The response rate was 0%, and the disease control rate was 80.0%. The OS rates were 58.3% at 6 months and 35.0% at 1 year after starting this treatment. The median OS after treatment initiation was 6.3 months (95% *CI* 2.97–9.56) (Fig 2(A)). The PFS rates since treatment initiation were 20.0% at 6 months and 20.0% at 1 year. The median PFS after this treatment was 3.7 months (95% *CI* 1.69–5.71) (Fig 2(B)). There was no significant difference in the OS and PFS since this treatment was started between patients who underwent no chemotherapy and those who underwent GC (*P* = 0.625, 0.334).

**Fig 3:**
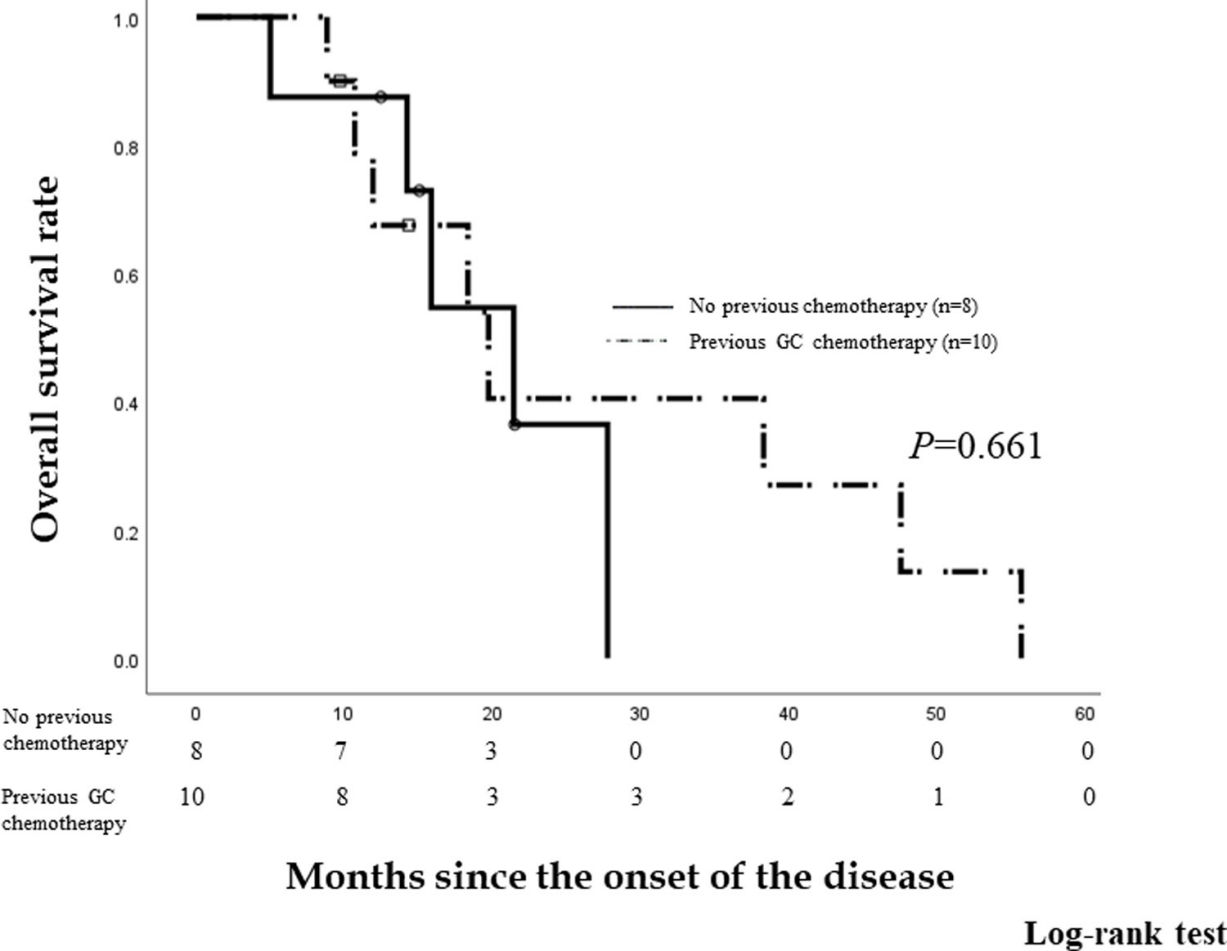
Overall survival (OS) rates of patients who did not receive previous chemotherapy and patients who received GC chemotherapy. The OS rates since disease onset were 87.5% and 90.0% at 6 months, 87.5% and 67.5% at 1 year, and 36.5% and 40.5% at 2 years in patients who did not receive previous chemotherapy and those who received GC chemotherapy, respectively. The median OS since disease onset was 21.4 months (95% *confidence interval* [*CI]* 13.7–29.18) and 19.7 months (95% *CI* 9.84–29.63), respectively. There was no significant difference in the OS since disease onset between patients who underwent no chemotherapy and those who underwent prior GC chemotherapy (*P =* 0.661).

We compared the OS rates of eight patients who did not have previous chemotherapy and ten patients who received prior GC chemotherapy since disease onset (Fig 3). The OS rates since disease onset were 87.5% and 90.0% at 6 months, 87.5% and 67.5% at 1 year, and 36.5% and 40.5% at 2 years, respectively. The median OS since disease onset is 21.4 months (95% *CI* 13.7–29.18) and 19.7 months (95% *CI* 9.84–29.63), respectively. There was no significant difference in the OS since disease onset between patients who underwent no chemotherapy and those who underwent prior GC chemotherapy (*P =* 0.661).

**Fig 4:**
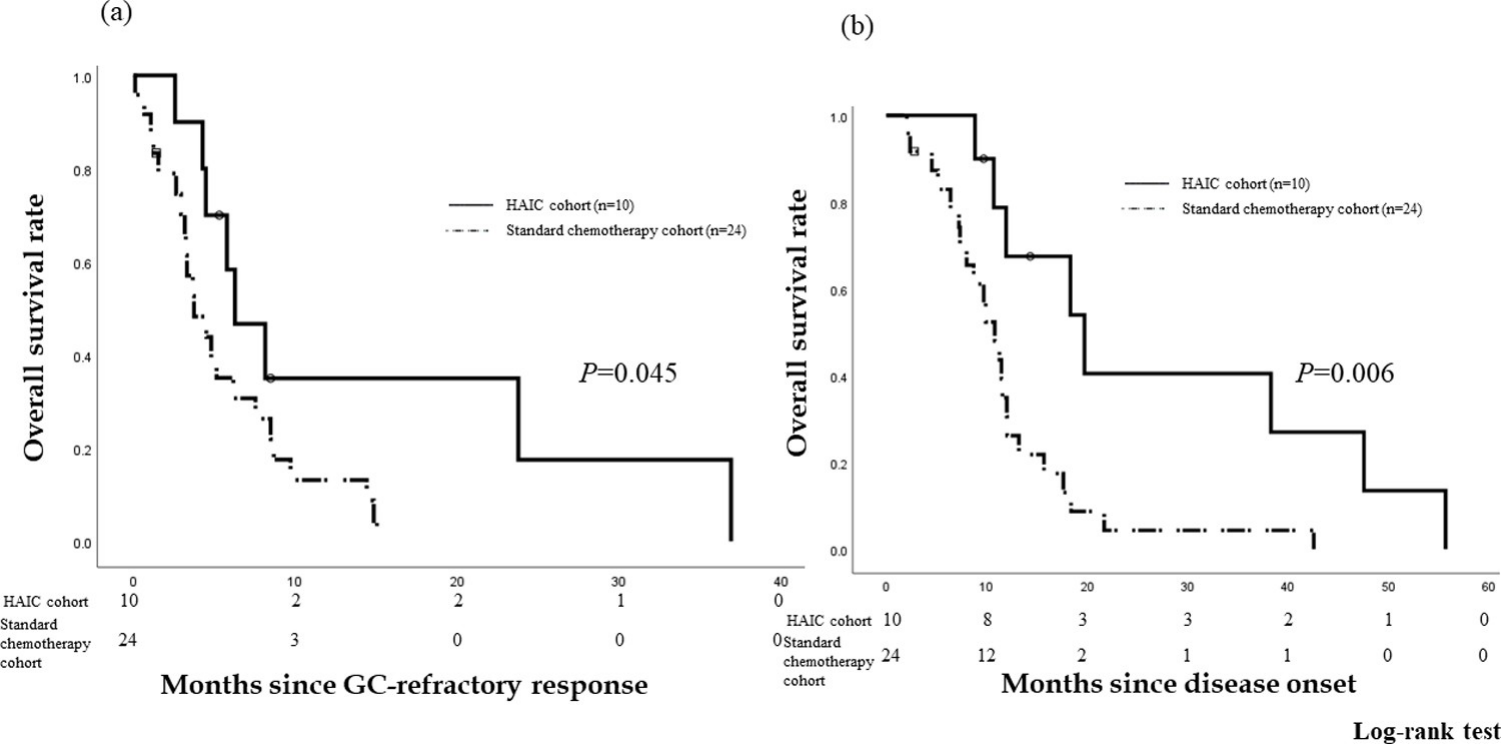
Overall survival (OS) rates of patients who received HAIC (HAIC cohort) vs. standard chemotherapy (standard chemotherapy cohort). Since the tumors were determined to be refractory to GC therapy, the OS rates were 58.3% and 35.1% at 6 months and 35.0% and 8.8% at 1 year for the HAIC and standard chemotherapy cohorts, respectively. The median OS after being refractory to GC was 6.3 months (95% *confidence interval* [*CI*] 2.97–9.56) and 3.7 months (95% *CI* 1.87–5.60), respectively. The OS of the HAIC cohort was better than that of the standard chemotherapy cohort (*P* = 0.045). Since disease onset, the OS rates were 67.5% and 30.6% at 1 year and 40.5% and 0% at 2 years, respectively. The median OS after the disease onset was 19.7 months (95% *CI* 9.84–29.6) and 10.8 months (95% *CI* 8.36–13.2), respectively. The OS of the HAIC cohort was better that of the standard chemotherapy cohort since the disease onset (*P* = 0.006).

We compared the OS rates of ten patients who underwent HAIC using GEM-FP and 24 patients who received standard chemotherapy due to GC-refractory tumors (Fig 4). Since the tumors were determined to be refractory to GC therapy, the OS rates were 58.3% and 35.1% at 6 months and 35.0% and 8.8% at 1 year for the HAIC and standard chemotherapy cohorts, respectively. The median OS after being refractory to GC was 6.3 months (95% *CI* 2.97–9.56) and 3.7 months (95% *CI* 1.87–5.60), respectively. The OS of the HAIC cohort was better than that of the standard chemotherapy cohort (*P* = 0.045) (Fig 4(A)). Since disease onset, the OS rates were 67.5% and 30.6% at 1 year and 40.5% and 0% at 2 years, respectively. The median OS after the disease onset was 19.7 months (95% *CI* 9.84–29.6) and 10.8 months (95% *CI* 8.36–13.2), respectively. The OS of the HAIC cohort was better than that of the standard chemotherapy cohort since the disease onset (*P* = 0.006) (Fig 4(B)).

**Fig 5:**
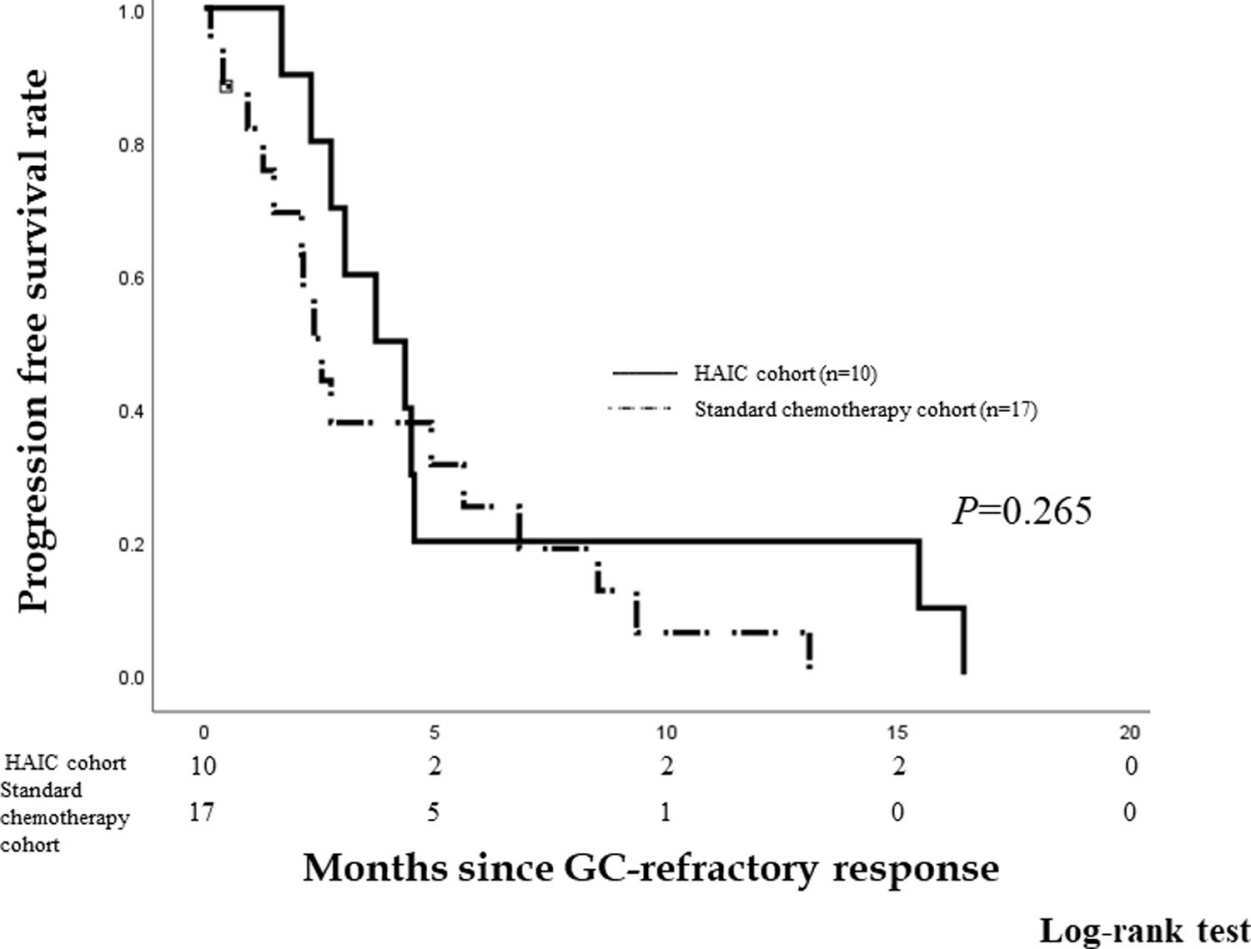
Progression free survival (PFS) rates of the HAIC and standard chemotherapy cohorts. The PFS rates were 20.0% and 25.2% at 6 months and 20.0% and 6.3% at 1 year, respectively. The median PFS was 3.7 months (95% *CI* 1.69–5.71) and 2.5 months (95% CI 1.76–3.31), respectively. There was no significant difference in PFS since found to be refractory to GC therapy between the HAIC and standard chemotherapy cohorts (*P* = 0.265).

Among the patients in standard chemotherapy cohort, 17 patients underwent other chemotherapy after being refractory to GC treatment. There were 15 patients who received S-1 and two patients who received gemcitabine. We compared the PFS of ten patients who underwent HAIC using GEM-FP and 17 patients who underwent standard chemotherapy since found to be GC-refractory. The PFS rates are 20.0%, and 25.2% at 6 months and 20.0% and 6.3% at 1 year in patients who underwent HAIC using GEM-FP and those who underwent standard chemotherapy, respectively. The median PFS is 3.7 months (95% *CI* 1.69–5.71) and 2.5 months (95% *CI* 1.76–3.31), respectively. There is no significant difference in PFS since determined as GC-refractory between the HAIC and standard chemotherapy cohorts (*P* = 0.265) (Fig 5).

### Adverse effects

Adverse effects were evaluated in 18 patients who received HAIC using GEM-FP in the HAIC cohort and 24 patients who received systemic GC in the standard chemotherapy cohort. Table 3 summarizes the patients’ adverse effects. Among the patients who received HAIC using GEM-FP, grade 3 or 4 toxicities (defined according to the Common Terminology Criteria for Adverse Events [15]) were found in six patients with anemia (33.3%), four patients with thrombocytopenia (22.2%), one patient with leukopenia (5.6%), and one patient with elevated bilirubin level (5.6%). Among the patients who received systemic GC chemotherapy, grade 3 or 4 toxicities were found in six patients with anemia (25%), six patients with thrombocytopenia (25%), three patients with leukopenia (12.5%), and two patients with elevated bilirubin level (8.3%). There was no difference in the incidence of side effects between the HAIC and systemic GC cohorts, except for leukopenia. Anemia may have had a relationship with the disease progression. Hepatic artery occlusion occurred in one patient (5.6%) in the HAIC cohort; however, it was not life-threatening.

**Table 3:** Adverse effects of hepatic arterial infusion chemotherapy using gemcitabine, cisplatin, and 5-fluorouracil vs systemic gemcitabine-cisplatin combination therapy.

Adverse effects	HAIC-GEM-FP (n = 18)		GC (n = 24)		
	Any grade	Grade 3 or 4	Any grade	Grade 3 or 4	*P*
**Leukopenia**	2 (11.1)	1 (5.6)	11 (45.8)	3 (12.5)	0.049
**Anemia**	12 (66.7)	6 (33.3)	19 (79.2)	6 (25)	0.396
**Thrombocytopenia**	5 (27.8)	4 (22.2)	11 (45.8)	6 (25)	0.368
**Hyperbilirubinemia**	4 (22.2)	1 (5.6)	2 (8.3)	2 (8.3)	0.114
**AST/ALT level elevation**	4 (22.2)	0 (0)	4 (16.7)	0 (0)	0.650
**Fever**	4 (22.2)	0 (0)	3 (12.5)	0 (0)	0.403
**General fatigue**	3 (16.6)	0 (0)	2 (8.3)	0 (0)	0.409
**Nausea/vomiting**	1 (5.6)	0 (0)	2 (8.3)	0 (0)	0.729
**Gastric ulcer**	1 (5.6)	0 (0)	0 (0)	0 (0)	0.243
**Artery occlusion**	1 (5.6)	–	0 (0)	-	0.243

Data are expressed as n (%)

AST, alanine aminotransferase; ALT, aspartate transaminase; GC, gemcitabine plus cisplatin combination therapy

## Discussion

In the present study, HAIC using GEM-FP was shown to be effective in patients with advanced ICC. In particular, this treatment was effective in patients who did not undergo chemotherapy. Similarly, it was also effective in patients who received GC and were refractory to it. This treatment had high disease control rates and consequent survival benefits for patients who received prior chemotherapy.

The prognosis of advanced BTC is poor. Park et al. [16] reported a median OS of 3.9 months in patients who were not treated with surgery, chemotherapy, or radiotherapy. The two main clinical trials for BTC (ABC-02 and BT22 trials) showed that GC yields a greater survival benefit than gemcitabine monotherapy in patients with advanced BTC [3, 4]. GC has been established as the standard chemotherapy for patients with advanced BTC in many countries. In our study, the median OS for patients who did not undergo chemotherapy (20.6 months) was longer than that for patients who participated in these two main clinical trials (ABC-02: 11.7 months, BT22: 11.2 months). The median PFS for the patients who underwent no previous chemotherapy (8.1 months) was also longer than the median PFS of these two main clinical trials (ABC-02: 8.0 months, BT22: 5.8 months). This treatment was effective for the patients who did not undergo chemotherapy.

Second-line chemotherapy has not been established for patients with advanced BTC, as compared to several other cancers in which second-line or subsequent chemotherapies have been established. Some previous clinical trials reported on the second-line treatment for patients with advanced BTC refractory to GC [17–19]. Although HAIC using GEM-FP has a low response rate, this treatment has the highest disease control rate in these clinical trials (FOLFOX4: 62.2%, BGJ398: 75.4%, Pembrolizumab: 35.3%). In Japan, S-1 is often used as a second-line chemotherapy for advanced BTC. In our study, there is no significant difference in PFS since GC-refractory response between the HAIC and standard chemotherapy cohorts (*P* = 0.265). Moreover, eight patients (80%) in the present study underwent two or three systemic chemotherapies before undergoing HAIC using GEM-FP. On the other hand, there were a few patients who underwent more than three chemotherapies among the standard chemotherapy cohort. Therefore, the PFS of this treatment was favorable.

The median OS of the patients who received HAIC using GEM-FP after GC since disease onset was 19.7 months. This result was more favorable in patients with advanced BTC who received only GC [3, 4]. Kim et al. [20] reported that post-progression survival without chemotherapy after refractory response to GC was 2.5 months. In Japan, a few doctors recommend the best supportive care for patients with advanced BTC refractory to GC. In fact, the OS of the HAIC cohort was better than that of the standard chemotherapy cohort since the GC-refractory response and disease onset (*P* = 0.045, 0.006). Although HAIC using GEM-FP for patients with advanced ICC does not have a high response rate, this treatment has a high disease control rate and consequent survival benefit for patients who have undergone previous chemotherapy. Therefore, HAIC using GEM-FP should be added in the treatment for advanced ICC.

HAIC has been shown to be an effective treatment for advanced hepatocellular carcinoma in Asian countries, especially in Japan [5, 21]. Although HAIC is not a common treatment for patients with advanced ICC, some reports have shown that HAIC has offered superior tumor control rates compared with systemic chemotherapy [7, 22, 23]. Although ICC normally does not have a large supply of arterial blood from the hepatic artery, it is at least supplied by the arterial blood from the hepatic artery. In addition, we identified the artery supplying blood to the tumor from CT during hepatic arteriography. We confirmed the blood flow feeding the tumor by CT hepatic arteriography. We utilized all arteries supplying blood to the tumor using the System-i [14]. This modality enables the feeding artery to be used during every HAIC. Therefore, this technical procedure may have led to favorable results in this study. However, there were a few side effects observed in this study. HAIC has low toxicity to the whole body and is more tolerable than systemic chemotherapy. Particularly, patients who underwent several prior chemotherapies had no physical strength.

In this study, the patients who had undergone systemic GC underwent HAIC using gemcitabine and cisplatin. When patients were refractory to systemic GC, HAIC using GEM-FP was effective in eight of ten (80%) cases. Sawai et al. [24] reported that HAIC using the same drugs was effective in systemic chemotherapy-resistant colon cancer. However, the mechanism behind this phenomenon is unclear. High concentrations of anticancer agents in the tumor may be effective against resistant tumor cells. As a result, this treatment has high disease control rate in patients refractory to systemic GC.

To our knowledge, the present study is the first to present the results of HAIC using gemcitabine and cisplatin. Previous studies showed the effectiveness of HAIC using gemcitabine for ICC. According to Inaba et al., the median OS of patients who received HAIC using gemcitabine was 12.9 months [11]. The majority of patients in the study conducted by Inaba et al. had not received any previous chemotherapy. In our study, the results for patients who had not received any chemotherapy was more favorable than those reported by the previous report. In addition, Ghiringhelli et al. reported that the median OS of patients who received HAIC using gemcitabine and oxaliplatin as the second-line treatment was 20.3 months [12]. In our study, eight patients (80%) underwent two or three systemic chemotherapies before undergoing HAIC using GEM-FP. Importantly, hepatic arterial infusion in combination with gemcitabine may be more effective than HAIC-gemcitabine monotherapy.

This study has some limitations. Our study was a retrospective analysis at two centers, which limits the validity of the results. Additionally, HAIC required a radiologist’s technical support during the procedure; thus, it is not necessarily something that any person can administer. In the HAIC cohort, some patients had previously received systemic GCF chemotherapy. Moreover, we did not compare the effect of HAIC using gemcitabine, cisplatin, and 5-fluorouracil to HAIC using either gemcitabine or 5-fluorouracil. Going forward, multicenter, prospective, randomized-controlled studies are required to investigate the effectiveness of HAIC using GEM-FP for patients with advanced ICC.

## Conclusion

HAIC using GEM-FP may be effective for patients with advanced ICC compared to other chemotherapies. In particular, this therapy was effective for patients who did not undergo chemotherapy. Further, this treatment was identically effective for patients who underwent GC and were refractory to it. Although this treatment did not have high response rates, it had high disease control rates and consequent survival benefits. This treatment may be useful as a therapeutic option for patients with advanced ICC.
